# *Alphacoronavirus* in a Daubenton’s Myotis Bat (*Myotis daubentonii*) in Sweden

**DOI:** 10.3390/v14030556

**Published:** 2022-03-08

**Authors:** Olivia Wesula Lwande, Therese Thalin, Johnny de Jong, Andreas Sjödin, Jonas Näslund, Magnus Evander, Frauke Ecke

**Affiliations:** 1 Department of Clinical Microbiology, Umeå University, 901 85 Umea, Sweden; olivia.lwande@umu.se; 2 Department of Wildlife, Fish, and Environmental Studies, Swedish University of Agricultural Sciences, 901 83 Umea, Sweden; teth0001@stud.slu.se (T.T.); frauke.ecke@slu.se (F.E.); 3 Swedish Biodiversity Centre (CBM), Department of Urban and Rural Development, Swedish University of Agricultural Sciences, 750 07 Uppsala, Sweden; johnny.de.jong@slu.se; 4 Division of CBRN Defence and Security, Swedish Defence Research Agency FOI, 906 21 Umea, Sweden; andreas.sjodin@foi.se (A.S.); jonas.naslund@foi.se (J.N.)

**Keywords:** coronavirus, bats, Myotis daubentonii, Pipistrellus pygmaeus, Sweden

## Abstract

The ongoing COVID-19 pandemic has stimulated a search for reservoirs and species potentially involved in back and forth transmission. Studies have postulated bats as one of the key reservoirs of coronaviruses (CoVs), and different CoVs have been detected in bats. So far, CoVs have not been found in bats in Sweden and we therefore tested whether they carry CoVs. In summer 2020, we sampled a total of 77 adult bats comprising 74 *Myotis daubentonii*, 2 *Pipistrellus pygmaeus*, and 1 *M. mystacinus* bats in southern Sweden. Blood, saliva and feces were sampled, processed and subjected to a virus next-generation sequencing target enrichment protocol. An *Alphacoronavirus* was detected and sequenced from feces of a *M. daubentonii* adult female bat. Phylogenetic analysis of the almost complete virus genome revealed a close relationship with Finnish and Danish strains. This was the first finding of a CoV in bats in Sweden, and bats may play a role in the transmission cycle of CoVs in Sweden. Focused and targeted surveillance of CoVs in bats is warranted, with consideration of potential conflicts between public health and nature conservation required as many bat species in Europe are threatened and protected.

## 1. Introduction

In the last decade, coronaviruses (CoVs) have gained interest due to the severity of outbreaks they cause [1]. Severe acute respiratory syndrome coronavirus-2 (SARS-CoV-2) is causing the COVID-19 pandemic, and CoVs are also responsible for the SARS-CoV outbreak of 2002 and the continuing epidemic of Middle East respiratory syndrome coronavirus (MERS-CoV), first detected in 2012 [2,3]. It is believed that CoVs are capable of spilling over from wildlife reservoirs to humans because they circulate in multiple hosts, including bats, birds and humans. CoVs are large, single-stranded, positive-sense RNA viruses in the family *Coronaviridae*, order *Nidovirales*. CoVs are grouped into four distinct genera, in which *Alpha*- and *Betacoronavirus* infect mammals, while *Gamma*- and *Deltacoronavirus* mainly are found in birds [4]. Metagenomics studies have revealed the presence of *Alpha*- and *Betacoronavirus* in many different bat species [5]. Alpha-CoVs include 14 viral genera and 19 species [6]. Two human alpha-CoVs, HCoV-229E and HCoV-NL63, have been identified [7,8]; they are prevalent worldwide, and usually cause mild cold-like symptoms [8,9]. The majority of alpha-CoVs are bat CoVs, and are hosted by numerous bat species, but they also infect other animals such as pigs, dogs, cats and rodents [6]. SARS-CoV-2 belongs to the *Betacoronavirus* genus, and has been shown to form a distinct clade in lineage B of the subgenus *Sarbecovirus*, together with a bat-derived SARS-CoV-like strain bat-SL-CoVZC45 [10].

In Fennoscandia (Denmark, Norway, Sweden and Finland), there is limited surveillance on bat CoVs. Nevertheless, in Denmark, alphacoronaviruses (alpha-CoVs) were found in the feces of five bat species (*Myotis (M) daubentonii*, *M. dasycneme*, *M. nattereri*, *Pipistrellus pygmaeus* and *Eptesicus serotinus*) sampled at the entrances of their hibernacula [11,12]. The CoV sequences found in *M. daubentonii* resembled those found in conspecifics in Germany and United Kingdom [11]. In Finland, alpha-CoVs were detected in feces of *M. daubentonii* and *M. brandtii* and a beta-CoV in that of *Eptescius nilssonii*, and the CoV sequences found in *M. daubentonii* resembled those of conspecifics in Germany and Spain [13]. However, CoVs have not yet been detected in bats in Sweden.

In Sweden, 19 bat species are known to occur, of which all are protected and 12 are red-listed [14]. Daubenton’s myotis (*M. daubentonii*) is a Eurasian bat occurring in large parts of Europe and in a band stretching along the middle parts of western Russia. In south–central Sweden, it is one of the most common bat species. It often forages close to the water surface along streams and above lakes, which makes it easy to find and to trap. In Sweden, it forms colonies in buildings and in hollow trees. The comparatively high prevalence of CoVs in *M. daubentonii* in previous studies in Fennoscandia [11,12,13], along with their commonness and their hunting strategy that facilitates trapping in summer, makes them suitable study species to screen for CoVs in bats in Sweden.

Here, we studied if CoVs occur in bats sampled in southern Sweden, and to which degree any found CoVs are related to CoVs found in neighboring countries.

## 2. Materials and Methods

### 2.1. Ethical Considerations

The trapping and sampling of bats was approved by the Animal Ethics Committee in Uppsala (Ref. nr. 5.8.18-01713/2020) and by the Swedish Environmental Protection Agency (Ref. nr. NV-02918-20), and all applicable institutional and national guidelines for the use of animals were followed.

### 2.2. Bat Sampling

**Figure 1:**
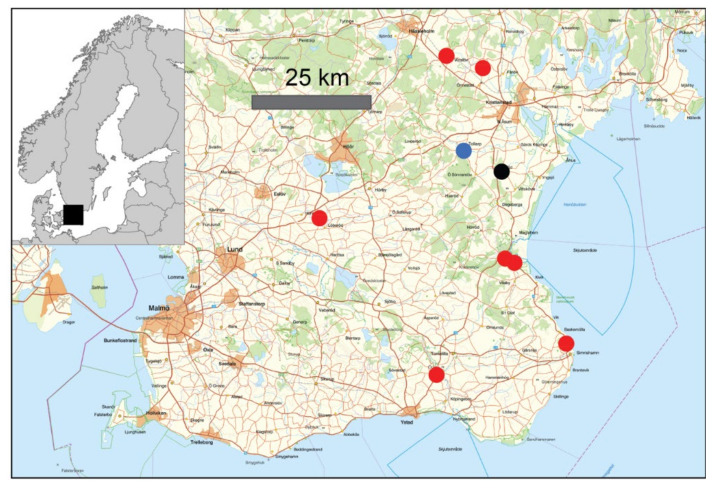
Spatial location of the nine bat trapping localities in southern Sweden. The black square in the map of Fennoscandia indicates the study area. Coloration of circles indicates the species that were trapped. Red circles: only Daubenton’s myotis (*Myotis daubentonii*), blue circle: *M. daubentonii* and Soprano pipistrelle (*Pipistrellus pygmaeus*), black circle: *M. daubentonii*, *Pipistrellus pygmaeus* and whiskered myotis (*M. mystacinus*).

Bat sampling was performed on 13–24 July 2020 in southern Sweden. Bats were captured at nine stream localities (Figure 1 and Table 1) using mist nets mounted perpendicular to small streams from sunset until midnight. Captured bats were immediately disentangled from the net and kept in a cotton bag until further handling. Specimens were determined to species level and weighed. The bats were divided into two age groups, juveniles and adult bats > 1 year old, based on the fusion in the epiphyseal gap of the finger joints. We collected saliva, blood and feces from each specimen; however, feces were only collected from 75 bats since 2 bats did not produce any feces in the cotton bags. Briefly, saliva samples were obtained by gently swabbing the mouth with a FLOQSwab^®^. The swab was immediately placed in virus transport medium (UTM 1 mL Minitip Flocked Transport & Preserv. Medium) and stored on dry ice until transport to the laboratory. About 100 µL of blood was collected from the interfemoral vein in the uropatagium and stored dry on a Nobuto blood filter strips (Advantec, Toyo Roshi Kaisha Ltd., Tokyo, Japan), which were stored at room temperature until further processing. Feces from each bat were collected from the cotton bag and stored in a 2 mL cryovial which was stored on dry ice. All samples were transported to the laboratory and stored at −80 °C pending processing.

### 2.3. Sample Processing of Saliva, Feces and Blood from Bats

All samples were prepared and analyzed separately. The saliva swabs in the stored tubes were thawed on ice, vortexed for 15 s and the homogenous mixture used for RNA extraction. The respective bat feces were placed in 2 mL screw-capped tubes containing three 2 mm steel beads (AB Nino Lab, Upplands Väsby, Sweden) and 1000 μL of Hanks buffer (HBSS) supplemented with 500 IU penicillin, 500 µg streptomycin and 3 µg amphotericin. Homogenization was performed using FastPreps 120 (Q-BIOgene, Irvine, CA, USA) at 6.5 m/s for 20 s. The homogenates were further filtered using a 0.2 µm syringe. For the blood samples, each filter paper was cut carefully using a sterile surgical blade (Swann-Morton^®^, Sheffield, UK), placed in a 1.5 mL microcentrifuge tube and eluted with 500 µL phosphate-buffered saline containing 0.05% Tween and 0.08% sodium azide. The tubes containing the filters were incubated overnight on a shaking rack at 4 °C. The next morning, the eluate was pipetted into new microcentrifuge tubes and centrifuged for 2 min at 10,500× *rcf* to free the supernatants from any debris which might have been formed during the elution. All samples were stored at −80 °C pending the RNA extraction.

### 2.4. RNA Extraction

Extraction of viral RNA, from saliva, feces and blood samples was performed with the QIAmp^®^ Viral RNA Mini Kit (QIAGEN, Hilden, Germany) according to the manufacturer’s protocol (Spin Protocol). A 140 μL measure of each sample was used as a sample volume and eluted in a final volume of 60 μL, collected in a 1.5 mL sterile microcentrifuge tube and stored at −80 °C.

### 2.5. Pan-Viral Panel Protocol

The current study employed a next-generation sequencing target enrichment protocol specific for viruses known as the Twist Comprehensive Viral Research Panel (CVRP) (Twist Biosciences, San Francisco, CA, USA), which covers reference sequences for 3153 viruses, including 15,488 different strains. The method has been applied in screening of patient samples for infectious viruses, as in the case of respiratory viral co-infections with rhino- and influenzavirus in patients confirmed to have SARS-CoV-2 [15]. The CVRP has been designed to be applicable within the Illumina TruSeq RNA Library Prep for Enrichment and TruSeq RNA Enrichment workflows.

Briefly, the RNA was converted to cDNA using ProtoScript II First Strand cDNA Synthesis Kit (E6560S) and New England Biolab’s Random Primer 6 (S1230S). The NEBNext Ultra II Non-Directional RNA Second Strand Synthesis kit (E6111S) was subsequently used to convert single-stranded cDNA to dsDNA. Illumina TruSeq-compatible libraries were then generated using the CVRP with Enzymatic Fragmentation (PN 101059 and 100401) and Unique Dual Indices (UDI) (PN 101307). Libraries were ultimately generated at a viral titer of 91.3 ng/μL. Hybridization capture was performed using the CVRP (PNs 103545, 103547, 103548) and the Twist Standard Target Enrichment workflow. Approximately 9.6 ng/μL of library was used in each 16 h hybridization capture reaction. Following enrichment, libraries were sequenced with 75 bp paired-end reads on the Illumina MiSeq platform, using a MiSeq Reagent v3 150-cycles kit.

### 2.6. Taxonomic Classification and Virus Genome Assembly of Metagenomic Neads

Generated sequence reads were classified using Kaiju [16] to give a profile of potential virus species in enriched samples. Sequence reads were also assemblies using Megahit [17] and Trinity [18] and contigs longer than 1000 bp were kept and polished using Pilon [19]. Remaining contigs were annotated using Prokka [20] and characterized using Checkv [21] and Virsorter [22]. Predicted virus sequences were then further annotated and confirmed using NCBI BLAST+ [23]. To analyze the similarity of the genomic organization of identified alpha-CoV with other alpha-CoV, we used Simplot analysis (see also [24] employing R packages ggmsa and gggenes.

### 2.7. Phylogenetic Analyses of Alphacoronavirus Sequences

The virus genome sequence obtained from the study was aligned to the publically available alpha-CoV sequences downloaded from the Virus Pathogen Resource (VIPR) [25], complemented with metagenomic assembled corona sequences from Denmark [12]. A total of 1328 sequences were aligned using MAFFT version 7 [26] with Middle East respiratory syndrome coronavirus (Genbank accession KJ713299) as an outgroup. Bootstrapped maximum likelihood phylogenetic trees were calculated using IQTREE [27] with ultrafast bootstrap approximation (1000 replicates) [28] and visualized using iTOL [29]. A subset of 52 genome sequences was selected from the complete dataset to generate another detailed phylogeny based on closely related sequences. All software were installed using Bioconda [30] through the workflow manager Snakemake [31]. Sequence data are available at NCBI BioProject PRJNA795992 and GenBank provisional accession number OK663601.

## 3. Results

### 3.1. Bat Sampling and Species Identification

A total of 77 bats were sampled from nine different locations in southern Sweden (Figure 1 and Table 1). *M. daubentonii* was the targeted species with 74 specimens. In addition, we trapped two soprano pipistrelle (*Pipistrellus pygmaeus*) and one whiskered myotis (*M. mystacinus*) (Figure 1 and Table 1). Of the 77 bats, 76 were adults and 1 was a juvenile, and 28 of the trapped bats were males and 49 females.

### 3.2. Screening of Bat Samples for Virus Detection

The bats were sampled to detect viruses, and 16 samples were randomly selected for next-generation sequencing using the CVRP (10). Using this technique, a CoV sequence (alpha-CoV, GenBank provisional accession number OK663601, 21,882 nucleotides) spanning the near complete genome was obtained from 1 of 16 samples (6.3%), the feces of a female adult *M. daubentonii* collected from Tollarp (Table 1).

### 3.3. Phylogenetic and Genetic Analysis

**Figure 2:**
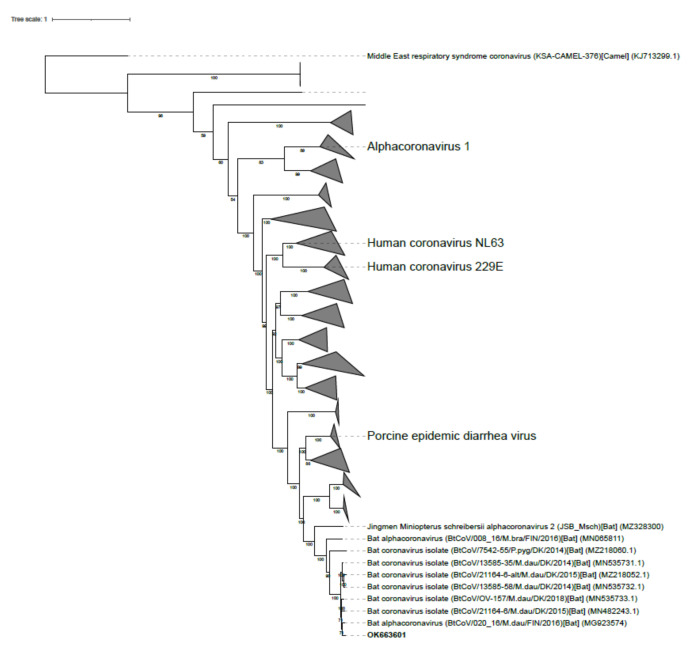
Phylogenetic relationships between the nucleotide sequence of the alpha-CoV detected in the study (OK663601 in bold) and 1328 other strains. The Middle East respiratory syndrome coronavirus (MERS-CoV) (KJ713299.1) was used as an outgroup. The differently shaped triangles symbolize clusters of related sequences.

**Figure 3:**
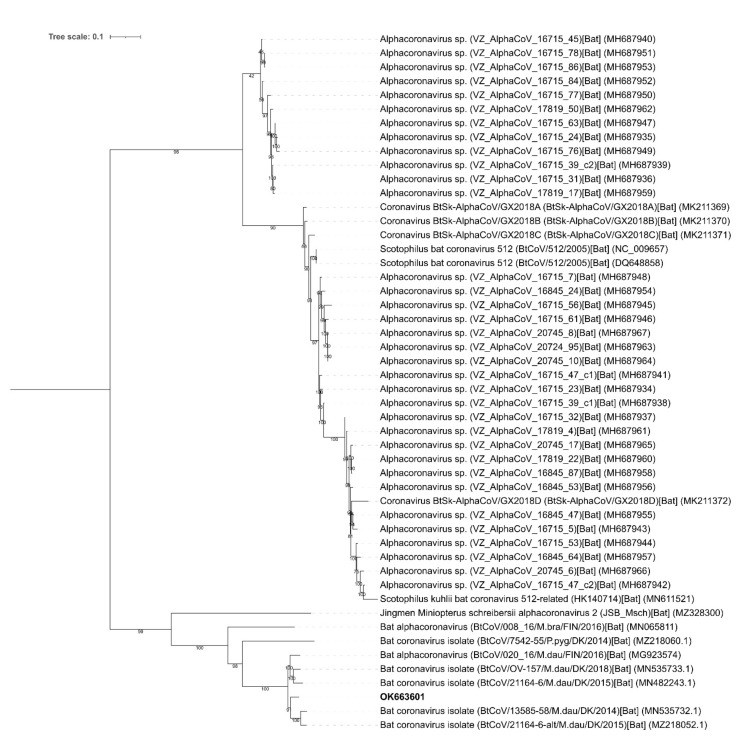
Phylogenetic relationships between the nucleotide sequence of the alpha-CoV detected in the study (OK663601 in bold) and 52 other strains. The Middle East respiratory syndrome coronavirus (MERS-CoV) (KJ713299.1) was used as an outgroup (Figure 2).

**Figure 4:**
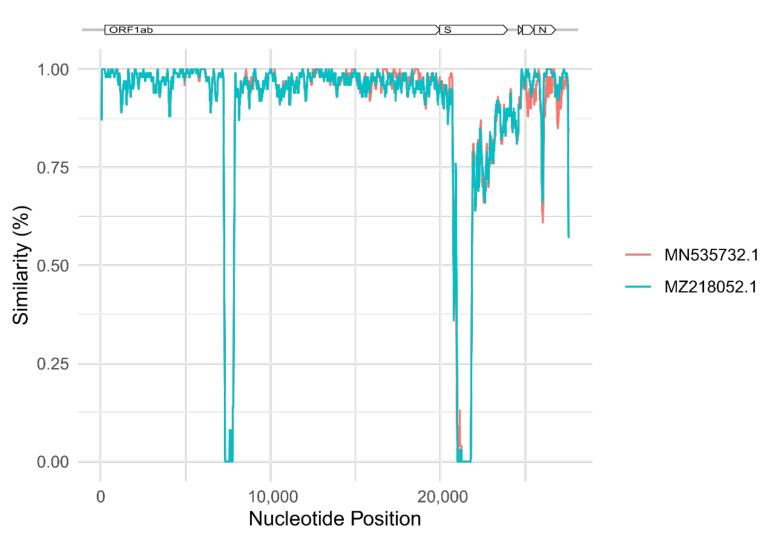
Simplot analysis of OK663601 with the two most closely related viruses (MN535732.1 and MZ2118052.1).

The nucleotide sequence was compared to all other publically available alpha-CoV genomes (1328 sequences) and visualized in the phylogenetic tree shown in Figure 2. A representative subset of 52 sequences were selected from the complete dataset and the novel virus sequence from the Swedish bat had high identity (95%) to both Danish and Finnish alpha-CoV strains detected in *M. daubentonii* (Figure 3). In addition, the phylogenetic analysis indicated that the study strain OK663601 found in Sweden was most closely related to alpha-CoV strains from Denmark and Finland, detected in 2014–2018, all from *M. daubentonii* (Figure 3). The genomic organization of the novel alpha-CoV was similar to that of other alpha-CoVs (Figure 4).

## 4. Discussion

This was the first report of coronavirus presence in bats in Sweden, while it has previously been found in Denmark, Finland and other European countries [11,12,13]. For detection and sequencing of the coronavirus, we used a method comprising next-generation sequencing with virus-specific targets. This technique is promising, and it has been successfully used for virus screening previously (10). The sequence comparisons showed that the virus was an alpha-CoV within the *Coronaviridae* family. Two features of alpha-CoVs that sets them aside from other coronaviruses are a unique type of nsp1 and the presence of commonly shared accessory gene (designated ORF3 in most alpha-CoV species) for a dispensable alpha-CoV membrane protein (αmp) [32]. The sequence had the features of alpha-CoV, although there were sequence gaps (in ORF1 and S) that can be completed, but this was not within the scope of this study. The novel Swedish alpha-CoV sequence formed a cluster, mainly with other alpha-CoVs isolated from *M. daubentonii*. The sequence was highly similar to full-length viral genome sequences from *M. daubentonii* from Denmark [12] and Finland [13]. Within the cluster, there were relatively closely related alpha-CoV sequences from other bat species, one from a Danish alpha-CoV detected in *Pipstrellus pygmaeus* in Denmark, 2014, and another from a *Miniopterus shreibersii* in China, 2021 [33] (Figure 3). These sequences are part of a larger cluster, which includes the *Pedacovirus* subgenus (as defined by ICTV). The finding suggests the presence and possible circulation of bat-borne CoVs in Sweden. Moreover, the high similarity between our alpha-CoV strain and the Finnish and Danish strains in *M. daubentonii* suggests low geographical barriers. *M. daubentonii* may be the predominant species linked with CoV in Sweden, Denmark and Finland [11,12,13].

Bats can move over large distances, but the migration behavior differs considerably between species [34]. *M. daubentonii* is considered to be a facultative migrant, moving short to middle-range distances between roosting and hibernation sites. In autumn, the species often forages at sea along the Swedish coast [35], and it is most likely that a number of individuals move between Sweden and Denmark/Germany, and between Sweden and Finland. The longest distance of movement recorded for a *M. daubentonii* bat so far is 150 km [34]. In Sweden, *M*. *daubentonii* is found in the south and central parts where the shortest distance between Sweden and Denmark is 3 km, between Sweden and Germany 70 km, and between Sweden and Finland 40 km. Investigation of the genetic structure of *M. daubentonii* in Britain has shown that there is no migration barrier between Britain and continental Europe [36]. *M. daubentonii* is highly gregarious and gathers in colonies in winter, and multiple individuals can roost together, e.g., in tree hollows. The *M. daubentonii* specimens trapped in our study might therefore have been in contact with conspecifics in Denmark and or Finland. The bat’s sociality in combination with its movement patterns likely increases pathogen transmission [37] and might explain the high nucleotide sequence homology of alpha-CoVs in *M. daubentonii* in Fennoscandia, as well as for other bat-borne pathogens [38]. The phylogenetic clustering (Figure 2) indicates that the alpha-CoVs may be geographically restricted, i.e., that alpha-CoVs from Northern Europe are clustered together. Notably, irrespective of the reservoir bat species, the viruses clustered relatively close together (Figure 2) [39,40].

One of 16 bats (6.3%) in our study was alpha-CoV-positive. Given the sociality of *M. daubentonii* and the previously detected prevalence of the virus in this bat species in two studies in Finland (19%) [13] and Denmark (23%) [11], we might expect that more Swedish bats would carry alpha-CoVs. At this point, we do not have an explanation for the fact that only one bat was positive in our study, although the low number of samples may in part explain the prevalence. In the future, a proper prevalence study comprising more samples from bats needs to be performed to obtain a better picture of the prevalence. Surveillance studies on bat species distribution and abundance in Sweden indicate that *M. daubentonii* is the most common species [41], implying that if they are indeed involved in alpha-CoV transmission, they may contribute to the transmission of the virus over large distances. *M. daubentonii* demonstrates temporal sexual segregation [42,43,44,45,46,47,48]. This phenomenon is not clearly understood, but due to only one positive sample in our study, we are unfortunately unable to shed further light on the role of sociality for pathogen transmission in *M. daubentonii*. Future studies should study the prevalence of CoVs in bats in Sweden with special focus on the role of migration and sociality for pathogen transmission. Focused and targeted surveillance of CoVs in bats is indeed warranted, with consideration of potential conflicts between public health and nature conservation required as many bat species in Europe are threatened and protected.

## 5. Conclusions

A CoV belonging to the *Alphacoronavirus* genus was discovered in a specimen of *M. daubentonii* in Sweden by using a pan-viral panel combined with next-generation sequencing. Bats may play a role in the transmission cycle of CoV in Sweden.

## Figures and Tables

**Table 1:** Summary of the nine trapping localities as well as number of specimens per bat species (Md: *Myotis daubentonii*, Pp: *Pipistrellus pygmaeus*, Mm: *Myotis mystacinus*), sex and age.

Name of Locality ^1^	Stream ^1^	Coordinates ^1^		Species			Sex		Age	
		Longitude	Latitude	Md	Pp	Mm	Males	Females	Adults	Juveniles
Tollarp	Vramsån	13.975	55.930	16	1	0	1	16	16	1
Ängsbo	Verkaån	14.155	55.721	7	0	0	1	6	7	0
Bosarp	Verkaån	14.124	55.728	1	0	0	1	0	1	0
Allevadsmölla	Nybroån	13.905	55.505	3	0	0	0	3	3	0
Rålambsdal	Vinne å	14.033	56.087	12	0	0	6	6	12	0
Rövarekulans naturreservat	Bråån	13.497	55.794	6	0	0	6	0	6	0
Everöd	Mjöån	14.105	55.892	5	1	1	5	2	7	0
Simrishamn	Tommarpaån	14.335	55.570	18	0	0	5	13	18	0
Vinslöv	Vinne å	13.909	56.108	6	0	0	3	3	6	0

^1^ See also Figure 1.

## Data Availability

Not applicable.
